# Enhancement of Berberine Hypoglycemic Activity by Oligomeric Proanthocyanidins

**DOI:** 10.3390/molecules23123318

**Published:** 2018-12-14

**Authors:** Haoyue Zhang, Xueping Wang, Ting Wang, Kaixian Chen, Heyao Wang, Qi Jia, Yiming Li

**Affiliations:** 1 School of Pharmacy, Shanghai University of Traditional Chinese Medicine, 1200 Cailun Road, Shanghai 201203, China; 13817075605@163.com (H.Z.); xueping723@163.com (X.W.); 0000008001@shutcm.edu.cn (K.C.); 2 Institute of Interdisciplinary Integrative Medicine Research, Shanghai University of Traditional Chinese Medicine, Shanghai 201203, China; 3 Shanghai Institute of Materia Medica, Chinese Academy of Sciences, Shanghai 201203, China; zhenwojingcaihaha@126.com

**Keywords:** oligomeric proanthocyanidins, P-glycoprotein, hypoglycemic effect, berberine, pharmacokinetics

## Abstract

This study investigated the possible enhancement of berberine’s (BB) hypoglycemic activity by oligomeric proanthocyanidins (OPCs) and its underlying mechanism. The hypoglycemic activity of the studied compounds was evaluated in diabetic *db*/*db* mice. The cellular uptake and efflux of BB with or without OPCs were investigated using Caco-2 intestinal cells. A pharmacokinetic study of BB and OPCs was performed in Sprague Dawley (SD) mice by oral administration of the study compounds. Liquid chromatography–tandem mass spectrometry (LC–MS/MS) was employed to determine the cellular efflux, retention, and the serum concentrations of the compounds. The results revealed that OPCs considerably potentiated the hypoglycemic efficacy of BB in diabetic *db*/*db* mice. In the in vitro experiments, OPCs significantly inhibited the efflux and increased the uptake of the P-glycoprotein (P-gp) substrate rhodamine-123 (R123) and BB in Caco-2 intestinal cells. Moreover, OPCs substantially reduced the expression of P-gp in Caco-2 cells. The inhibition of BB efflux by OPCs was translated into the improved pharmacokinetics in vivo. When co-administered, OPCs obviously increased the average maximum concentration of BB in mice. In summary, this study demonstrated that combination of BB with OPCs could significantly improve the pharmacokinetics and hypoglycemic efficacy of BB, which is valuable for future exploration of the combination of BB and OPCs as oral hypoglycemic agents.

## 1. Introduction

Diabetes mellitus (DM) is a chronic metabolic disorder characterized by hyperglycemia and hyperlipidemia. With the development of the social economy, DM, particularly type 2 diabetes mellitus (T2DM), has become a considerable threat to public health [1]. Although several classes of effective oral antihyperglycemic agents are currently available, including biguanides, sulfonylureas, thiazolidinediones, α-glucosidase inhibitors, dipeptidylpeptidase-4 inhibitors, and sodium glucose cotransporter 2 inhibitors, these medications frequently have varying adverse effects or limitations [2]. Therefore, searching for safer and more effective hypoglycemic drugs has become the focus of antidiabetic medication research.

Substantial evidence exists to support the use of traditional Chinese medicine (TCM) interventions for the treatment of DM. More importantly, these TCM treatments appear to cause significant reductions in DM-related complications beyond plasma glucose regulation [3,4,5]. A total of 86 natural medicines are commonly used in TCM preparations to treat T2DM and its complications [5]. Among these, Rhizoma coptidis—prepared from the rhizomes of *Coptis chinensis* (Franch) and also known as Huang Lian in Chinese—and its major constituent berberine (BB) have attracted considerable attention for their remarkable hypoglycemic and hypolipidemic activity [6,7,8,9]. The antihyperglycemic activity of BB was first reported in 1986 [10]. Moreover, BB has demonstrated positive effects in treating diabetic nephropathy [11], diabetic neuropathy [12], and diabetic cardiomyopathy [13]. Thus, BB is a promising natural-product candidate for novel antidiabetic drugs. However, its poor oral bioavailability (<1%) is a notable disadvantage [14]. BB is a substrate of P-gp, which pumps BB into the intestinal lumen and limits the intestinal absorption of BB [15,16]. Therefore, the efflux inhibition of P-gp may be an effective method for promoting the absorption and use of BB, thereby improving its therapeutic effect.

Jiaotaiwan (JTW) is a classical TCM prescription composed of Rhizoma coptidis and cinnamon (Rou Gui in Chinese) that is usually used in TCM to treat insomnia. JTW was first mentioned in the book “Han Shi Yi Tong” compiled 500 years ago in the Chinese Ming Dynasty [17]. Numerous clinical and laboratory studies have indicated that JTW is beneficial in the treatment of T2DM. JTW significantly improved hyperglycemia, hyperlipidemia, and hepatic lipid accumulation in diabetic rats. Its possible mechanisms are related to the downregulation of acetyl coenzyme A carboxylase (ACC) and fatty acid synthase protein expressions alongside the upregulation of AMP-activated protein kinase (AMPK), phosphorylated-ACC, and glucose transporter 4 (GLUT4) protein expressions [18,19]. The presence of cinnamon water extract may affect the antidiabetic ability and pharmacokinetics of BB in JTW [20]. The therapeutic effect of JTW has also been reported to be more favorable than the use of its components individually for treating T2DM in rats [18,19,20].

Cinnamon is a genus of the Lauraceae family, some of whose members have been used as a spice, flavoring agent, preservative, and pharmacologic agent in several cultures for centuries [21,22]. Many studies have explored the beneficial effects of cinnamon for various ailments and their symptoms, such as diabetes, hyperlipidemia, arthritis, gastrointestinal disorders, Alzheimer disease, arteriosclerosis, and cancer [23,24,25,26]. Cinnamon polyphenols are mainly oligomeric proanthocyanidins (OPCs) and are regarded as the major antidiabetic components of cinnamon water extract [27,28,29]. Our previous studies have proved that OPC extracts from cinnamon exhibit clear hypoglycemic properties at the dosage of 200 mg/kg body weight [30,31]. OPCs have also been found to be P-gp substrates [32]. These findings suggest that some ingredients in cinnamon may affect the antidiabetic ability of BB in JTW in terms of pharmacodynamics or pharmacokinetics. Here, the possible enhancement of BB’s hypoglycemic activity by OPCs was investigated for the first time through pharmacokinetic in this study.

## 2. Results

### 2.1. OPCs Notably Potentiates the Hypoglycemic Effect of BB in Diabetic Mice

We investigated the hypoglycemic effect of the formula BB combined with OPCs in the diabetic mice. The male C57BLKS/J *db*/*db* and *db*/*m* diabetic mice were left untreated or orally treated with BB with or without OPCs. After treatment for 5 weeks, the mice were sacrificed. Fasting blood glucose (FBG) levels, oral glucose tolerance test (OGTT), intraperitoneal insulin tolerance test (IPTT), total triglyceride (TG), total cholesterol (TC), alanine transaminase (ALT), and aspartate transaminase (AST) were assayed. The Guide for the Care and Use of Laboratory Animals was strictly complied, and the animal experiment protocols were approved by the Institutional Animal Committee of Shanghai Institute of Materia Medica [Permit number: SYXK(Shanghai)2013-0049].

**Figure 1:**
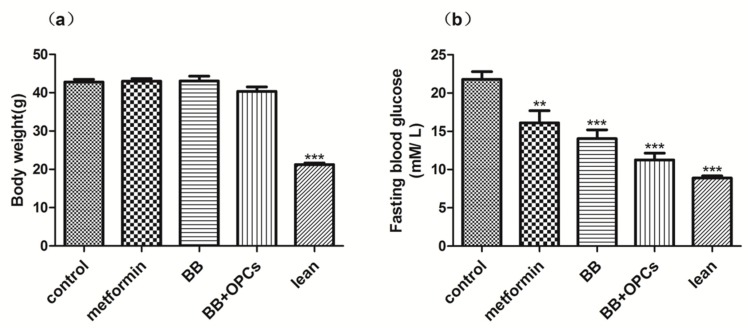
Effects of compounds on body weight and fasting glucose levels in *db*/*db* mice. Male C57BLKS/J *db*/*db* and *db*/*m* diabetic mice were left untreated or orally treated with metformin at 150 mg/kg, BB at 200 mg/kg, or BB at 200 mg/kg plus OPCs at 60 mg/kg, respectively. After treatment for 4 weeks, body weight (**a**) and fasting glucose (**b**) levels were measured. Data are presented as mean ± SEM of eight mice in each group. ** p < 0.01, *** p < 0.001 vs. untreated control.

Findings revealed no significant difference in body weight among the 4 groups after 4 weeks of repeated oral administration (Figure 1a). Thus, the administration of metformin (150 mg/kg), BB (200 mg/kg), and BB (200 mg/kg) plus OPCs (60 mg/kg) had no significant effect on *db*/*db* mice obesity. However, the *db*/*db* mice treated with BB with or without OPCs exhibited significantly lower FBG than did the nontreated *db*/*db* mice (Figure 1b). Moreover, the group treated with BB plus OPCs exhibited markedly higher lowering of FBG values, by 48% (*p* < 0.001 vs. control), than did the group treated with BB only, which lowered FBG by 35% (*p* < 0.001 vs. control).

**Figure 2:**
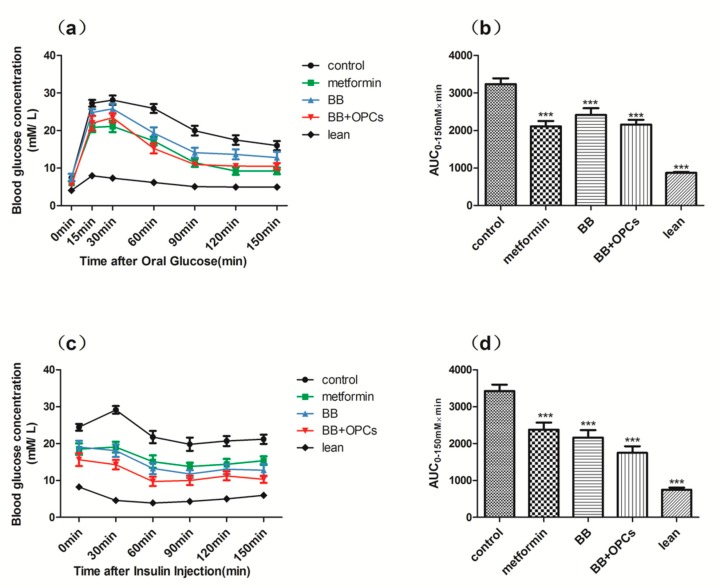
Effects of the 5 weeks administration of compounds in the (**a**) OGTT and (**c**) IPTT in *db*/*db* mice with diabetes. (**a**) After 14 h of fasting, 0.35 g of glucose per kilogram of body weight was gavaged into mice and calculated as 0 min. Blood glucose levels were measured at the indicated times shown in the graph. (**c**) After 6 h of fasting, blood glucose concentrations were measured at the indicated time following intraperitoneal injection of 1 IU of insulin per kilogram of body weight. AUCs for (**b**) OGTT and (**d**) IPTT are shown in the graph. Data are presented as mean ± SEM of eight mice in each group. *** p < 0.001 vs. untreated control.

Similar results were obtained in the OGTT and IPTT. As shown in Figure 2, the area under the curve (AUC) for the OGTT and IPTT in the *db*/*db* mice was significantly lower in the mice treated with metformin and BB with or without OPCs than in the nontreated *db*/*db* mice (*p* < 0.001 vs. control). Furthermore, the AUC for the OGTT and IPTT of the *db*/*db* mice in the group treated with BB plus OPCs was slightly lower than that of the *db*/*db* mice in the group treated with BB alone, although neither exhibited a significant difference (*p* > 0.05 vs. BB alone). These results indicated that the combination of BB and OPCs improved the glucose tolerance and insulin sensitivity of the *db*/*db* mice more effectively than BB treatment alone.

**Figure 3:**
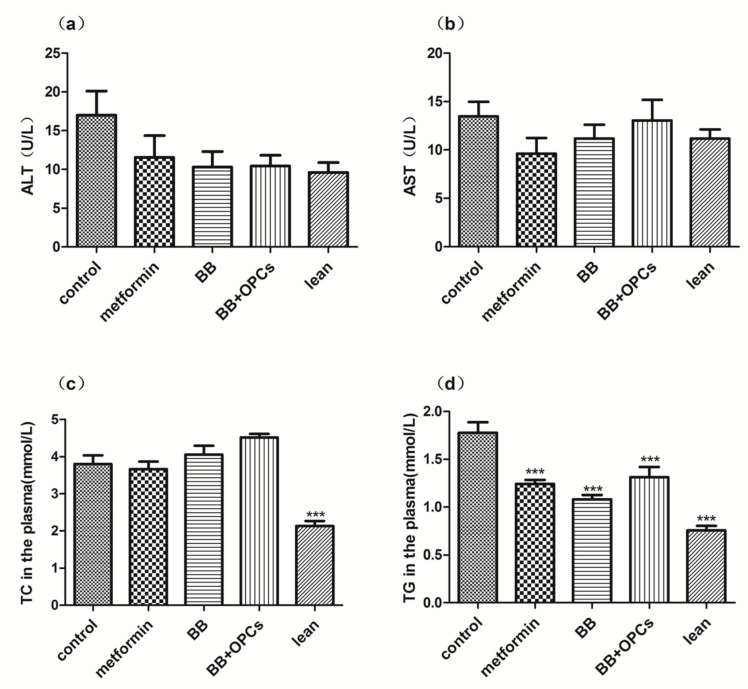
Effects of compounds on ALT, AST, TC, TG levels in *db*/*db* mice. The *db*/*db* mice were treated with the vehicle, 150 mg/kg of metformin, 200 mg/kg of BB and 200 mg/kg of BB plus 60 mg/kg of OPCs for 5 weeks. Then, the (**a**) ALT in the plasma. (**b**) AST in the plasma. (**c**) TC in the plasma. (**d**) TG in the plasma were measured. Data are presented as mean ± SEM of eight mice in each group. *** p < 0.001 vs. untreated control.

In addition to hypoglycemic activity tests, several biochemical indexes in serum were also investigated. As shown in Figure 3, compared with the control group, the serum levels of ALT and AST in each medicated group exhibited no significant difference, indicating that BB with or without OPCs did not cause hepatotoxicity. The TC level in the serum of each medicated group also exhibited no significant difference (*p* > 0.05), whereas the TG level in the serum of each medicated group was markedly decreased (*p* < 0.001 vs. control). These data indicated that the combination of BB and OPCs could ameliorate the diabetic symptoms of the *db*/*db* diabetic mice by promoting lipid metabolism.

### 2.2. In Vitro Cytotoxicity and Reverse Effect of BB and OPCs on Caco-2 Cells

**Figure 4:**
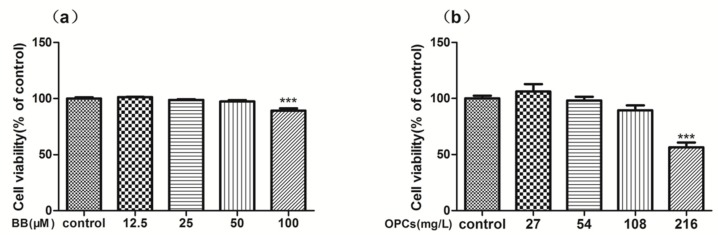
Cytotoxicity and reversing activity of BB and OPCs by MTT assay in Caco-2 cells. Cells were treated with various concentrations of BB (**a**) and OPCs (**b**) for 24 h for toxic determination. Data are presented as mean ± SEM of three independent experiments. *** p < 0.001 vs. control.

To measure the cytotoxicity of BB and OPCs on Caco-2 cells by using the methylthiazolyldiphenyl-tetrazolium bromide (MTT) assay, the cells were exposed to different concentrations of BB and OPCs for 24 h. The results illustrated in Figure 4a revealed no toxic effect on Caco-2 cells under the concentrations of BB ranging from 12.5 to 50 μM for 24 h. This result indicated that this concentration range of BB could be used for the next experiment. The result illustrated in Figure 4b revealed no toxic effect on Caco-2 cells under concentrations of OPCs ranging from 27 to 108 mg/L for 24 h; however, significant toxicity was demonstrated when 216 mg/L of OPCs was added for 24 h. Based on these results, 25 μM of BB and 108 mg/L of OPCs were used in the forthcoming experiments (here, we set the OPC concentration as 100 μM; according to the molecular weight of its main ingredients (864), 100 μM of OPCs can be converted to 108 mg/L).

### 2.3. OPCs Enhanced Intracellular Accumulation of R123 in Caco-2 Cells

**Figure 5:**
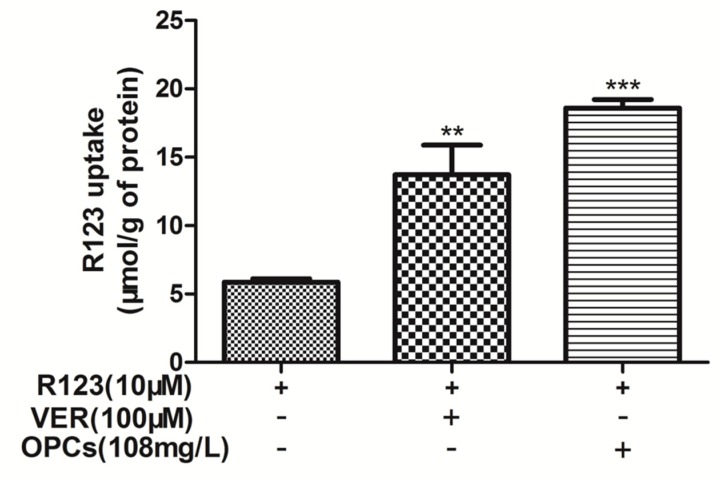
The effects of OPCs on R123 accumulation in Caco-2 cells were investigated at 108 mg/L. Verapamil (100 μM) was used as positive control. Data are presented as mean ± SEM of three independent experiments. ** p < 0.01, *** p < 0.001 vs. control.

To investigate whether OPCs can inhibit the efflux function of P-gp, the intracellular accumulation of R123, a classical substrate of P-gp, was measured in Caco-2 cells. The cells were treated with 108 mg/L of OPCs and 10 μM of R123. The P-gp inhibitor verapamil (100 μM) with 10 μM of R123 was used as a positive control. Compared with the control group that was administered with R123 alone, the intracellular accumulation of R123 significantly increased for both combinations (Figure 5). However, the enhancement effect of 108 mg/L of OPCs on R123 was 35% higher than that of 100 μM of verapamil. These data indicated that OPCs can inhibit P-gp efflux and enable the retention of P-gp substrates in Caco-2 cells.

### 2.4. OPCs Increased the Uptake of BB in Caco-2 Cells

**Figure 6:**
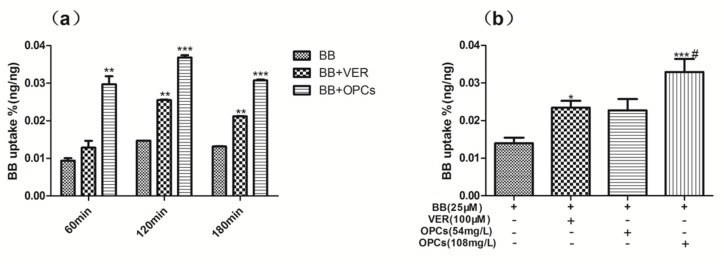
OPCs increased the uptake of BB in Caco-2 Cells. Caco-2 intestinal cells were treated with 25 μM of BB and BB with OPCs at 108 mg/L for different time (**a**) or BB with OPCs at 54 mg/L or 108 mg/L for 2 h (**b**). Verapamil (100 μM) was used as positive control, cells were harvested for determination of the uptake of BB. Quantities of BB in the cells were determined by LC–MS/MS. Data are presented as mean ± SEM of three independent experiments. * p < 0.05, ** p < 0.01, *** p < 0.001 vs. control (BB alone), ^#^ *p* < 0.05 108 mg/L of OPCs vs. verapamil.

As a substrate of P-gp, BB was poorly absorbed in the intestinal epithelium, which shows high levels of P-gp expression. To determine if OPCs affect the uptake of BB, Caco-2 cells were treated with BB (25 μM) and BB with OPCs at 108 mg/L for different lengths of time, and BB with OPCs was also tested at various concentrations for 2 h. The intracellular concentration of BB was determined through LC–MS/MS. As illustrated in Figure 6, OPCs promoted the absorption of BB in Caco-2 cells in a time and dose dependent manner. When co-administered with 108 mg/L of OPCs for 2 h, Caco-2 intestinal cells exhibited 200% higher uptake of BB (*p* < 0.001 vs. BB alone), and this was even higher than when co-administered with the positive control verapamil at 100 μM (*p* < 0.05 vs. verapamil). These data indicated that OPCs can increase the uptake of BB in Caco-2 cells.

### 2.5. OPCs Inhibited the Efflux of BB in Caco-2 Cells

**Figure 7:**
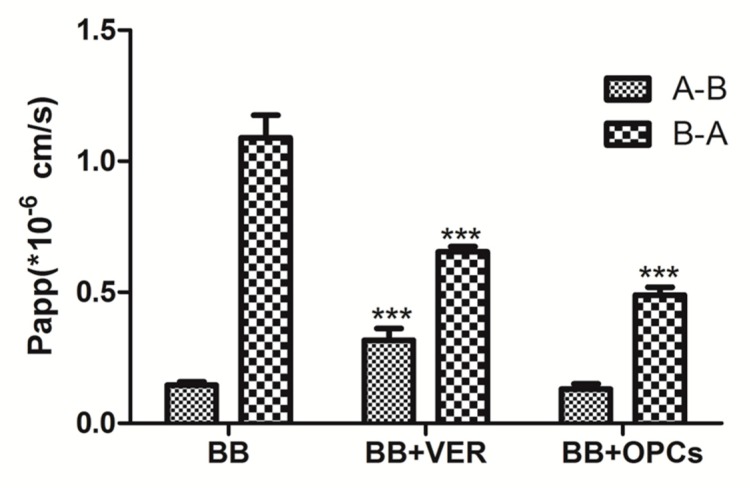
Effects of OPCs and verapamil on the apparent permeability (P_app_) of BB across Caco-2 monolayers. The apical to basolateral permeability values PappA-B and basolateral to apical permeability values PappB-A were determined as the slope of the linear portion of each transport-time profile. Data are presented as mean ± SEM of three independent experiments with 2 transwell inserts each. *** p < 0.001 vs. control (BB alone).

Whether P-gp inhibited the function of OPCs was also evaluated by measuring the transepithelial transport of BB across Caco-2 cell monolayers. Compared with the untreated control, all treatments significantly reduced the transport of BB in the basolateral (BL) to apical (AP) (B-A) direction (*p* < 0.001 vs. control); verapamil exhibited an enhancement of BB transport in the AP to BL (A-B) direction (*p* < 0.01 vs. control); and OPCs exhibited no apparent effect (Figure 7). The corresponding efflux ratios (R) are listed in Table 1; R values dropped significantly from 7.74 in the group treated with BB alone, to 4.10 in the group treated with BB and OPCs, and to 2.23 in the group treated with BB and verapamil. These data indicated that OPCs can inhibit the P-gp efflux of BB in Caco-2 cells.

### 2.6. OPCs Downregulated the Expression of P-gp in Caco-2 Cells

**Figure 8:**
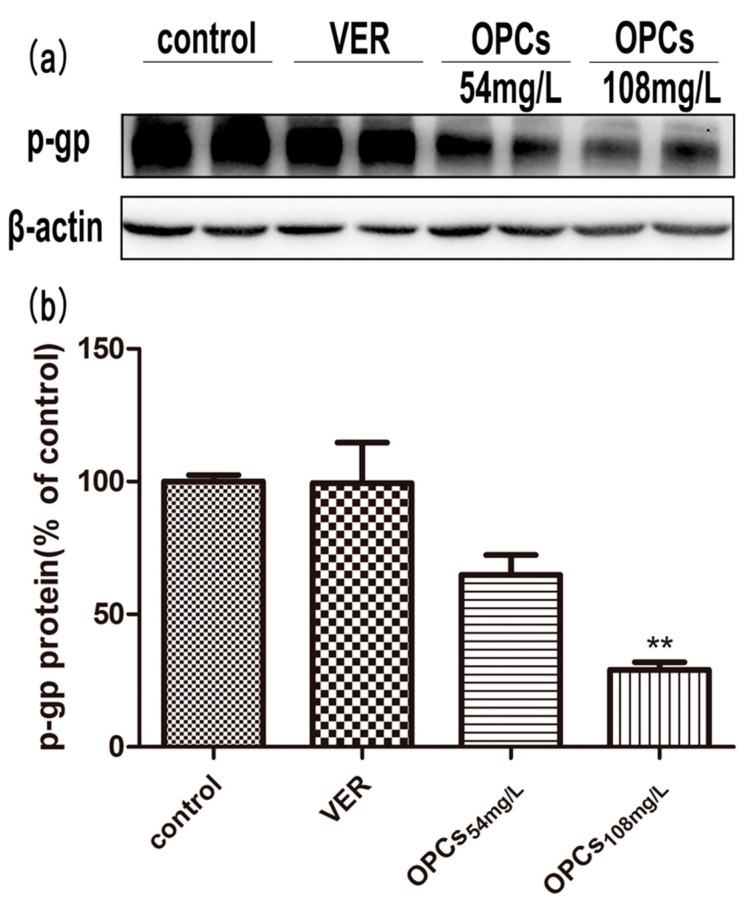
Effect of OPCs with different concentrations on P-gp expression for 24 h by Western blot. Representative blots were presented in the upper panel (**a**). Blots were scanned and quantified; the level of P-gp was normalized to that of β-Actin (ACTB) and plotted as percent of DMSO (**b**), which was designated as 100%. The quantitative data in histogram are presented as mean ± SEM of three independent experiments. ** p < 0.01 vs. control (DMSO).

To understand whether OPCs inhibited the function of P-gp by affecting its expression level, P-gp protein expression was quantified following 24-h treatment with various concentrations of OPCs. Compared with the control group, 54 mg/L of OPCs did not appear to have significant effects on P-gp expression (*p* > 0.05 vs. control), whereas 108 mg/L of OPCs reduced the P-gp protein level significantly (*p* < 0.01 vs. control) after exposure for 24 h (Figure 8). These results indicated that the protein expression level of P-gp decreased dose dependently after treatment with OPCs in Caco-2 cells.

### 2.7. OPCs Improve the Pharmacokinetics of BB in Mice

**Figure 9:**
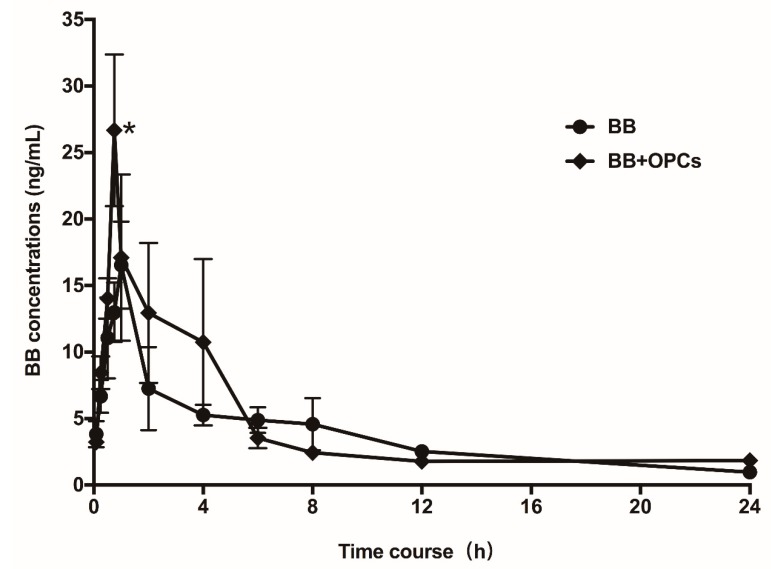
Pharmacokinetic Study. Male SD mice were orally administered with BB alone at 200 mg/kg, or 200 mg/kg of BB plus 120 mg/kg of OPCs, respectively. Blood samples were taken at 0, 0.083, 0.25, 0.5, 0.75, 1, 2, 4, 6, 8, 12, and 24 h after administration. Plasma concentrations of BB were determined by LC–MS/MS and plotted against time. Data are presented as mean ± SEM of six animals in each group. * p < 0.05 vs. that of the same time point.

To determine whether increased uptake and decreased cellular efflux by OPCs can improve the intestinal absorption and pharmacokinetic profiles of BB, the male SD mice were orally administered with BB alone (200 mg/kg) or BB plus 120 mg/kg OPCs. Subsequently, the plasma concentrations of BB were assayed at different time intervals. As illustrated in Figure 9, the average maximum concentration (Cmax) of BB when co-administered with OPCs was 29.7 ± 4.57 ng/mL, approximately 1.75-fold (*p* < 0.05) of that when only BB was administered (17 ± 2.96 ng/mL). The AUC_0–24_ of BB when co-administered with OPCs was 98.53 ± 21.54 ng·h/mL, approximately 1.24-fold of that when only BB was administered (78.98 ± 13.62 ng h/mL). These results suggested that the co-administration of OPCs improved the intestinal absorption and bioavailability of BB in mice. The Guide for the Care and Use of Laboratory Animals was strictly complied, and the animal experiment protocols were approved by the Institutional Animal Committee of Shanghai University of Traditional Chinese Medicine [Permit number: SZY201706022].

## 3. Discussion

The limitations of BB for clinical application as an antidiabetic drug are its poor availability and gastrointestinal side effects. Although synergistic hypoglycemic effects of BB with some compounds have been noted [33], the ideal synergistic drug for use with BB is yet to be determined. In this study, we investigated for the first time the synergistic hypoglycemic effect of BB with OPCs. Because OPCs have low toxicity with few side effects and have been developed for use in drugs (e.g., Crofelemer, a purified OPC extracted from the bark latex of *Croton lechleri*) [34], we believe they may be useful in the antidiabetic application of BB. 

Our findings suggested a synergistic hypoglycemic effect between BB and OPCs, which is likely a result of two notable aspects. As reported in the pharmacodynamics literatures, BB regulates glucose metabolism, possibly through increasing insulin sensitivity, which activates the AMPK pathway, inhibiting gluconeogenesis in the liver, stimulating glycolysis in the peripheral tissue cells, and increasing glucose transporter [35,36,37,38]. We previously reported that OPCs can regulate glucose metabolism mainly through protecting pancreatic β-cells by attenuating oxidative stress [39]. Thus, BB and OPCs may exert synergistic hypoglycemic effects through different mechanisms. BB is a substrate of P-gp, which can prevent the uptake of drugs from the gut and may interfere with the delivery of drugs to target tissues. This is the main reason for the poor oral bioavailability of BB. Procyanidins play a crucial role in drug interactions with P-gp substrates [40]. A study reported that procyanidine is a potent inhibitor of P-gp in the blood–brain barrier and could improve the therapeutic effects of some drugs on cerebral tumors [41].

With the administration of OPCs extract from cinnamon barks once daily for 4 weeks at the dosage of 200 mg/kg, we found that the fasting blood glucose of *db*/*db* mice was reduced and the oral glucose and insulin tolerances were improved. While at the dosage of 100 mg/kg, the fasting blood glucose of *db*/*db* mice shown no significant difference [42]. In this study, we demonstrated that the hypoglycemic efficacy of BB, which is a substrate of P-gp, was successfully potentiated by combination with 60 mg/kg of OPCs. We proved that the efflux of BB was blocked by OPCs and that the accumulation of BB increased considerably in Caco-2 intestinal cells that highly expressed P-gp. The effects of OPCs were translated into the improved pharmacokinetics of BB in animals. The addition of OPCs significantly improved the pharmacokinetic parameters of BB when co-administered. The oral bioavailability of BB in pharmacokinetic studies was well correlated with Caco-2 permeability in vitro.

Although we still cannot ascertain the mechanism underlying the effect of OPCs on P-gp, our findings indicated that OPCs can reduce not only P-gp efflux but also P-gp expression in Caco-2 intestinal cells; this result is supported by the literature, which indicates that procyanidin downregulated P-gp expression by inhibiting NF-κB activation and MAPK/ERK-mediated YB-1 activity in A2780/T cells [43]. In addition, we believe that OPCs are a competitive P-gp inhibitor, taking into account the current data which indicate that OPCs are transported paracellularly and are P-glycoprotein substrates [32]. This likely counteracts the upregulatory effect of BB on P-gp expression [44]. Additional experimental data are required to support this hypothesis.

BB and OPCs are the main constituents of Rhizoma coptidis and cinnamon, respectively. Based on TCM theory, Rhizoma coptidis has a “cold” nature and may cause gastrointestinal side effects when used for a long time. By contrast, the property of cinnamon is “warm”, and it can counterbalance the effect of the “cold” Rhizoma coptidis to reduce gastrointestinal discomfort. OPCs from *C. lechleri* (Crofelemer) have been used in the treatment of secretory diarrhea and diarrhea-predominant irritable bowel syndrome [34]. Whether OPCs can ameliorate the gastrointestinal side effects of BB through regulating the state of intestinal flora remains unclear and is worthy of consideration.

## 4. Materials and Methods

### 4.1. Preparation of OPCs

**Figure 10:**
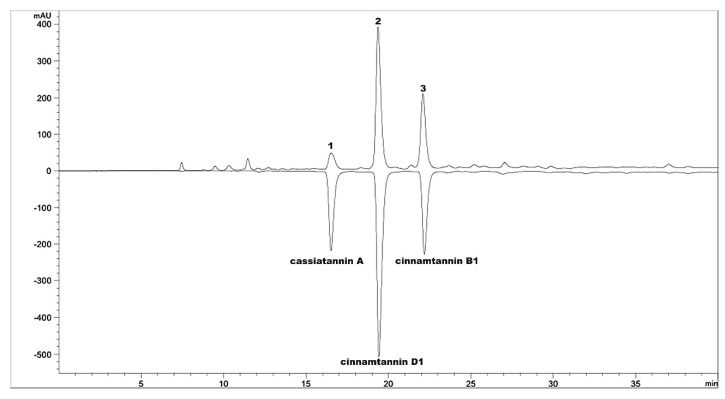
RP-HPLC chromatographic profile of OPCs detected at 280 nm. Peak 1 was A-type tetramer cassiatannin A, peaks 2 and 3 were A-type trimers, cinnamtannin D1 and cinnamtannin B1, respectively.

OPCs were isolated from the bark of *Cinnamomum cassia* (L.) as described previously [30] and then subjected to macro resin column chromatography and eluted with water and different ethanol concentrations. The 35% ethanol was removed in vacuo at 40 °C and subjected to freeze drying; the resulting extract consisted of OPCs. The principal component of OPCs were cassiatannin A, cinnamtannin D1, and cinnamtannin B1, as confirmed by high-performance liquid chromatography (HPLC: column, Agilent Extend C18 column, 5 μm, 4.6 × 250 mm; solvent system, acetonitrile with 0.1% phosphoric acid with gradient elution; flow rate, 1 mL/min; UV detection 280 nm; Agilent 1260, Agilent Technologies, Santa Clara, CA, USA). The HPLC chromatographic profile is illustrated in Figure 10. The total percentage of the three main OPCs was nearly 82% (7.1% cassiatannin A, 47.3% cinnamtannin D1, and 27.6% cinnamtannin B1).

### 4.2. Hypoglycemic Study in Diabetic Mice

Male C57BLKS/J *db*/*db* and *db*/*m* mice were obtained from the Model Animal Research Center of Nanjing University (Nanjing, China). The mice were housed in a temperature-and humidity-controlled room with free access to standard chow and water under a 12/12-h light/dark cycle. At 6 weeks of age, the diabetic *db*/*db* mice were randomly divided into 4 groups (n = 10 per group). Subsequently, one group of mice was gavaged once daily with the vehicle (0.5% carboxymethyl cellulose), one group was treated with BB (200 mg/kg body weight per day, diluted in 0.5% CMC-Na), one group was treated with BB (200 mg/kg of body weight) plus OPCs (60 mg/kg of body weight), and one group was treated with metformin (150 mg/kg of body weight) for 4 weeks. After 6 h of fasting, their blood glucose was monitored using blood from the tail vein and a glucometer (Accu-CHEK, Roche, IN, USA) every week. On Days 33 and 35 after drug administration, the mice were fasted for 12 and 6 h to perform an OGTT and IPTT, respectively. At the end of the experimental period, the mice’s blood was collected for further studies. Serum TG and TC levels were assayed using commercial enzyme assay kits (Rongsheng, Shanghai, China).

### 4.3. Cell Culture

Caco-2 cells were obtained from the American Type Culture Collection (Manassas, VA, USA). Caco-2 cells were cultured in Dulbecco’s modified Eagle medium (DMEM) containing 25 mM glucose supplemented with 10% fetal bovine serum. All cells at passage 24–43 were kept at 37 °C and 5% CO_2_ in humidified air.

### 4.4. Cell Viability Study

To investigate the effect of OPCs on cell viability, Caco-2 cells were seeded in 96-well plates. The cells were treated with different doses of compounds and incubated for 24 h. Cell viability was determined using the MTT assay. Briefly, the cells were incubated with the MTT reagent (0.5 mg/mL) at 37 °C for 4 h, and the absorbency at a wavelength of 492 nm was measured using FlexStation 3 (Molecular Devices, Sunnyvale, CA, USA). The percentage of optical density was calculated by comparing with that of the control group.

### 4.5. Intracellular Accumulation of Rhodamine 123

The effect of OPCs on the intracellular accumulation of rhodamine 123 (R123) was determined in Caco-2 cells. The cells treated with the most appropriate concentration of OPCs (108 mg/L) and R123 (10 μM) were incubated at 37 °C for 120 min. The P-gp inhibitor verapamil (100 μM) was used as a positive control. Subsequently, the cells were washed in phosphate-buffered saline (PBS) and lysed in 400 μL of 0.1% Triton X-100 solution through sonication. The intracellular concentrations of R123 were determined by measuring luminescence at an excitation wavelength of 485 nm and an emission wavelength of 530 nm by using FlexStation 3. The residual mixture was centrifuged at 12,000 rpm for 20 min, and the supernatant was quantitated using a (Bicinchoninic acid) BCA protein assay kit (Beyotime Biotechnology, Shanghai, China) for cell protein.

### 4.6. Cellular Uptake and Efflux Assays

#### 4.6.1. Uptake Studies of BB

Caco-2 cells were cultured at 37 °C in H-DMEM supplemented with 10% fetal bovine serum. Then, 24-well plates were used to seed the cells at a density of 2.5 × 10^4^ cells/cm^2^ for uptake experiments. The cells were then cultured for 2–3 days before use. Uptake experiments were initiated by incubating the plates with different concentrations of compound solutions and 25 μM BB for 2 h. The cells were washed twice with ice-cold PBS, and 0.4 mL of 0.1% Triton-100 diluted using Hank’s balanced salt solution (HBSS) was subsequently added. Ultrasound was conducted for 10 min at room temperature to obtain cell lysates. A total of 0.3 mL of the BB lysates was analyzed through LC–MS/MS, and 0.1 mL of the lysates was used for protein extraction and quantification. The intracellular BB concentration was normalized to the protein content and presented as ng/ng of protein.

#### 4.6.2. Transport Studies of BB

Caco-2 cells were seeded onto a 12-well polycarbonate membrane transwell plate with a pore size of 0.4 μm and a growth surface area of 1.12 cm^2^ at a density of 2.5 × 104 cells/cm^2^. Monolayers were employed at 21 days after seeding. To ensure the integrity of each cell monolayer, transepithelial electrical resistance was determined. Subsequently, cell monolayers were incubated with compounds in fresh incubation media from either the AP or BL side for designated time periods at 37 °C. The volume of incubation media on the AP and BL sides was 0.5 and 1.5 mL, respectively, and a 30 µL aliquot of the incubation solution was withdrawn at designated time points (60, 120, 180, and 240 min) from the receiver compartment and replaced with the same volume of a fresh prewarmed HBSS buffer.

BB concentrations were detected through LC–MS/MS. Apparent permeability (Papp) was calculated as follows:

Papp = (dQ/dt) × VA/(C_0_ × A) (1)

where dQ/dt is the permeability rate, A is the surface area of the monolayer, and C_0_ is the initial concentration of the drug.

### 4.7. Immunoblot Analysis

Cell lysates were prepared using RIPA lysis buffer containing 1 mM phenylmethylsulfonyl fluoride as a protease inhibitor. Protein concentrations were determined using a BCA estimation kit according to the manufacturer’s instructions. Cell protein (40 μg) was loaded onto each lane and separated through sodium dodecyl sulfate–polyacrylamide gel electrophoresis (6% separating gel). Separated proteins were transferred from the gel to the PVDF membrane. After blocking for 1 h with nonfat milk (5%, *w*/*v*) in Tris-buffered saline containing 0.1% Tween-20 (TBST), the primary antibody of P-gp (C219, Calbiochem, Darmstadt, Germany) at a dilution of 1:500 was added to TBST with 5% nonfat milk and then incubated with the membrane at 4 °C overnight. The membrane was washed before incubation with the corresponding secondary antibody at a dilution of 1:10,000 in the same buffer for 1.5 h at room temperature. Western blot signals were detected using an enhanced chemiluminescence detection agent.

### 4.8. Pharmacokinetic Study

Male SD mice (220 ± 5 g) were obtained from Bikai Experimental Animal Co., Ltd. (Shanghai, China) and maintained at the Shanghai University of TCM Laboratory Animal Center. After fasting overnight, the 2 groups of mice (6 in each group) were administered with 200 mg/kg of BB or 200 mg/kg of BB plus 120 mg/kg of OPC by using a gavage, respectively. Blood samples were collected through retro-orbital puncture and anticoagulated with heparin at 0, 0.083, 0.25, 0.5, 0.75, 1, 2, 4, 6, 8, 12 and 24 h after chemical administration. The plasma concentrations of BB were determined through LC–MS/MS.

### 4.9. LC–MS/MS Analysis

#### 4.9.1. Cellular Samples

The intracellular BB concentration was determined using an UltiMate 3000 Ultrahigh performance liquid chromatograph (UPLC) coupled to a Thermo Scientific TSQ Quantum Access MAX (Thermo Fisher, San Jose, CA, USA) mass spectrometer (UPLC–MS/MS) system, which consisted of a Surveyor UPLC pump with an online degasser, a Surveyor autosampler, and a TSQ Quantum triple quadrupole mass spectrometer equipped with electrospray ionization (ESI). A Waters Acquity UPLC HSS T3 (2.1 × 100 mm, 1.8 μm) column (Milford, MA, USA) was used for separation at 35 °C. The mobile phase consisted of 0.1% formic acid in water and 100% acetonitrile. The flow rate was 0.3 mL/min. The gradient program was from 30% to 40% acetonitrile for 0–0.5 min, 40% for 0.5 min, from 40% to 90% acetonitrile for 1.0–3.0 min, held at 90% for 1.0 min, and returned to initial conditions at 4.1 min to re-equilibrate for 2.0 min. Only the data from 0 min to 3.5 min were acquired through MS. The MS was operated in the positive ESI mode, and MS parameters were set as follows: spray voltage, 3.5 kV; sheath gas pressure, 30 arb; auxiliary gas pressure, 10 arb; vaporizer temperature, 350 °C; and capillary temperature, 350 °C. A precursor-to-product ion transition of *m*/*z* 336.0→320.2 for BB and *m*/*z* 356.4→192.2 for THP with tube lens voltage (TL) 82 V; collision energies (CE) 31 V for BB and TL 118 V; CE 27 V for THP were used for selected reaction monitoring. Data were analyzed using the Xcalibur 2.2 software (Version 2.2, Thermo Fisher Scientific Inc., Waltham, MA, USA, 2011).

#### 4.9.2. Serum Samples

The plasma concentrations of BB were determined using an AB Sciex Triple Quad 5500 (AB Sciex, Framingham, MA, USA) LC–MS/MS system with an ESI probe. A Waters Acquity UPLC HSS T3 (2.1 × 100 mm, 1.8 μm) column (Milford, MA, USA) was used for separation at 35 °C. The mobile phase consisted of 0.2% formic acid in water and 100% acetonitrile. The flow rate was 0.2 mL/min. The gradient program was 40% acetonitrile for 0–3.0 min, from 40% to 90% acetonitrile for 3.0–4.0 min, and returned to initial conditions at 4.1 min to re-equilibrate for 2.0 min. Only data from 0 to 4.0 min were acquired through MS. Analytes were quantified using the multiple-reaction monitoring mode. The precursor-to-product ion transition for BB and THP were the same as those aforementioned. For BB, they were as follows: CE, 39 V; declustering potential (DP), 100 V; and cell exit potential, (CEP) 18 V. For THP, they were as follows: CE, 27 V; DP, 119 V; and CEP, 16 V. Data were analyzed using the Analyst v1.5.2 software (Version 1.5.2, AB Sciex, Redwood City, CA, USA, 2011).

These assays were fully validated with reference to the US Food and Drug Administration Guidance for Industry Bioanalytical Method Validation, including selectivity, accuracy, precision, recovery, linearity, the matrix effect, and stability.

### 4.10. Statistical Analysis

All data are expressed as the mean ± SEM. The groups were compared using the one-way ANOVA analysis followed by Dunnett’s test and the Student’s *t* test for two groups. Differences were considered statistically significant at *p* < 0.05 or *p* < 0.01 or *p* < 0.001.

## 5. Conclusions

This study proved that combination with OPCs can improve the intestinal absorption and the in vivo hypoglycemic efficacy of BB. Our data suggested that formulas of BB combined with OPCs may be suitable for the treatment of chronic metabolic disorders. These results demonstrated the coordinated effect of OPCs on BB for the first time and provided a new direction for the development of hypoglycemic drugs based on the BB compound.

## Figures and Tables

**Table 1:** Effects of drugs on the transport of BB across Caco-2 cell monolayers.

	P_app_(A-B, ×10^−6^)	P_app_(B-A, ×10^−6^)	R = P_app_(B-A)/P_app_(A-B)
BB	0.146 ± 0.012	1.001 ± 0.049	7.74 ± 1.13
BB + VER	0.337 ± 0.040 ***	0.654 ± 0.020 ***	2.23 ± 0.41 ***
BB + OPCs	0.137 ± 0.021	0.489 ± 0.030 ***	4.10 ± 0.78 **

Data are presented as mean ± SEM of three independent experiments with two transwell inserts each. ** p < 0.01, *** p < 0.001 vs. control (BB alone).

## Abbreviations

BB	Berberine
OPCs	Oligomeric proanthocyanidins
SD mice	Sprague Dawley mice
LC–MS/MS	Liquid chromatography–tandem mass spectrometry
P-gp	P-glycoprotein
R123	Rhodamine-123
DM	Diabetes mellitus
T2DM	Type 2 diabetes mellitus
TCM	Traditional Chinese medicine
JTW	Jiaotaiwan
ACC	Acetyl coenzyme A carboxylase
AMPK	AMP-activated protein kinase
GLUT4	Glucose transporter 4
FBG	Fasting blood glucose
OGTT	Oral glucose tolerance test
IPTT	Intraperitoneal insulin tolerance test
TG	Total triglyceride
TC	Total cholesterol
ALT	Alanine transaminase
AST	Aspartate transaminase
AUC	Area under the curve
MTT	Methylthiazolyldiphenyl-tetrazolium bromide
DMEM	Dulbecco’s modified Eagle medium
HBSS	Hank’s balanced salt solution
HPLC	High-performance liquid chromatography
UPLC	Ultrahigh performance liquid chromatograph
ESI	Electrospray ionization
THP	Tetrahydropalmatine
TL	Tube lens voltage
CE	Collision energies
DP	Declustering potential
CEP	Cell exit potential
BL	Basolateral
AP	Apical
ACTB	β-Actin
BCA	Bicinchoninic acid

## References

[1] Zimmet P., Alberti K.G., Shaw J. (2001). Global and societal implications of the diabetes epidemic. Nature.

[2] Thrasher J. (2017). Pharmacologic Management of Type 2 Diabetes Mellitus: Available Therapies. Am. J. Cardiol..

[3] Rios J.L., Francini F., Schinella G.R. (2015). Natural Products for the Treatment of Type 2 Diabetes Mellitus. Planta Med..

[4] Seto S.W., Yang G.Y., Kiat H., Bensoussan A., Kwan Y.W., Chang D. (2015). Diabetes Mellitus, Cognitive Impairment, and Traditional Chinese Medicine. Int. J. Endocrinol..

[5] Li W.L., Zheng H.C., Bukuru J., De N.K. (2004). Natural medicines used in the traditional Chinese medical system for therapy of diabetes mellitus. J. Ethnopharmacol..

[6] Chang W., Chen L., Hatch G.M. (2015). Berberine as a therapy for type 2 diabetes and its complications: From mechanism of action to clinical studies. Biochem. Cell. Biol..

[7] Dong H., Wang N., Zhao L., Lu F. (2012). Berberine in the treatment of type 2 diabetes mellitus: A systemic review and meta-analysis. Evid.-Based Complement. Altern. Med..

[8] Pang B., Yu X.T., Zhou Q., Zhao T.Y., Wang H., Gu C.J., Tong X.L. (2015). Effect of Rhizoma coptidis (Huang Lian) on Treating Diabetes Mellitus. Evid.-Based Complement. Altern. Med..

[9] Pang B., Zhao L.H., Zhou Q., Zhao T.Y., Wang H., Gu C.J., Tong X.L. (2015). Application of berberine on treating type 2 diabetes mellitus. Int. J. Endocrinol..

[10] Chen Q.M., Xie M.Z. (1986). Studies on the hypoglycemic effect of Coptis chinensis and berberine. Acta Pharm. Sin..

[11] Wang F.L., Tang L.Q., Yang F., Zhu L.N., Cai M., Wei W. (2013). Renoprotective effects of berberine and its possible molecular mechanisms in combination of high-fat diet and low-dose streptozotocin-induced diabetic rats. Mol. Biol. Rep..

[12] Kim S.O., Kim H.J. (2013). Berberine ameliorates cold and mechanical allodynia in a rat model of diabetic neuropathy. J. Med. Food.

[13] Chang W., Zhang M., Meng Z., Yu Y., Yao F., Hatch G.M., Chen L. (2015). Berberine treatment prevents cardiac dysfunction and remodeling through activation of 5′-adenosine monophosphate-activated protein kinase in type 2 diabetic rats and in palmitate-induced hypertrophic H9c2 cells. Eur. J. Pharmacol..

[14] Chen W., Miao Y.Q., Fan D.J., Yang S.S., Lin X., Meng L.K., Tang X. (2011). Bioavailability study of berberine and the enhancing effects of TPGS on intestinal absorption in rats. AAPS PharmSciTech.

[15] Maeng H.J., Yoo H.J., Kim I.W., Song I.S., Chung S.J., Shim C.K. (2002). P-glycoprotein-mediated transport of berberine across Caco-2 cell monolayers. J. Pharm. Sci..

[16] Pan G.Y., Wang G.J., Liu X.D., Fawcett J.P., Xie Y.Y. (2002). The involvement of P-glycoprotein in berberine absorption. Pharmacol. Toxicol..

[17] Zhe Q., Sulei W., Weiwei T., Hongyan L., Jianwei W. (2017). Effects of Jiaotaiwan on depressive-like behavior in mice after lipopolysaccharide administration. Metab. Brain Dis..

[18] Hu N., Yuan L., Li H.J., Huang C., Mao Q.M., Zhang Y.Y., Lin M., Sun Y.Q., Zhong X.Y., Tang P., Lu X. (2013). Anti-Diabetic Activities of Jiaotaiwan in *db*/*db* Mice by Augmentation of AMPK Protein Activity and Upregulation of GLUT4 Expression. Evid.-Based Complement. Altern. Med..

[19] Huang Z., Xu X., Lu F., Wang N., Chen G., Zhao Y., Zou X., Wang K., Dong H., Xu L. (2013). Jiao tai wan attenuates hepatic lipid accumulation in type 2 diabetes mellitus. Evid.-Based Complement. Altern. Med..

[20] Chen G., Lu F., Xu L., Dong H., Yi P., Wang F., Huang Z., Zou X. (2013). The anti-diabetic effects and pharmacokinetic profiles of berberine in mice treated with Jiao-Tai-Wan and its compatibility. Phytomedicine.

[21] Gruenwald J., Freder J., Armbruester N. (2010). Cinnamon and health. Crit. Rev. Food Sci. Nutr..

[22] Ribeiro-Santos R., Andrade M., Madella D., Martinazzo A.P., Moura L.D.G., de Melo N.R., Sanches-Silva A. (2017). Revisiting an ancient spice with medicinal purposes: Cinnamon. Trends Food Sci. Technol..

[23] Hamidpour R., Hamidpour M., Hamidpour S., Shahlari M. (2015). Cinnamon from the selection of traditional applications to its novel effects on the inhibition of angiogenesis in cancer cells and prevention of Alzheimer’s disease, and a series of functions such as antioxidant, anticholesterol, antidiabetes, antibacterial, antifungal, nematicidal, acaracidal, and repellent activities. J. Tradit. Complement. Med..

[24] Hariri M., Ghiasvand R. (2016). Cinnamon and Chronic Diseases. Adv. Exp. Med. Biol..

[25] Kawatra P., Rajagopalan R. (2015). Cinnamon: Mystic powers of a minute ingredient. Pharm. Res..

[26] Rao P.V., Gan S.H. (2014). Cinnamon: A Multifaceted Medicinal Plant. Evid.-Based Complement. Altern. Med..

[27] Jia Q., Liu X., Wu X., Wang R., Hu X., Li Y., Huang C. (2009). Hypoglycemic activity of a polyphenolic oligomer-rich extract of Cinnamomum parthenoxylon bark in normal and streptozotocin-induced diabetic rats. Phytomedicine.

[28] Jiao L., Zhang X., Huang L., Gong H., Cheng B., Sun Y., Li Y., Liu Q., Zheng L., Huang K. (2013). Proanthocyanidins are the major anti-diabetic components of cinnamon water extract. Food Chem. Toxicol..

[29] Li R., Liang T., Xu L., Li Y., Zhang S., Duan X. (2013). Protective effect of cinnamon polyphenols against STZ-diabetic mice fed high-sugar, high-fat diet and its underlying mechanism. Food Chem. Toxicol..

[30] Chen L., Sun P., Wang T., Chen K., Jia Q., Wang H., Li Y. (2012). Diverse mechanisms of antidiabetic effects of the different procyanidin oligomer types of two different cinnamon species on *db*/*db* mice. J. Agric. Food Chem..

[31] Lu Z., Jia Q., Wang R., Wu X., Wu Y., Huang C., Li Y. (2011). Hypoglycemic activities of A- and B-type procyanidin oligomer-rich extracts from different Cinnamon barks. Phytomedicine.

[32] Zumdick S., Deters A., Hensel A. (2012). In vitro intestinal transport of oligomeric procyanidins (DP 2 to 4) across monolayers of Caco-2 cells. Fitoterapia.

[33] Shan Y.Q., Zhu Y.P., Pang J., Wang Y.X., Song D.Q., Kong W.J., Jiang J.D. (2013). Tetrandrine potentiates the hypoglycemic efficacy of berberine by inhibiting P-glycoprotein function. Biol. Pharm. Bull..

[34] Tradtrantip L., Namkung W., Verkman A.S. (2010). Crofelemer, an antisecretory antidiarrheal proanthocyanidin oligomer extracted from Croton lechleri, targets two distinct intestinal chloride channels. Mol. Pharmacol..

[35] Derosa G., Maffioli P., Cicero A.F.G. (2012). Berberine on metabolic and cardiovascular risk factors: An analysis from preclinical evidences to clinical trials. Expert Opin. Biol. Ther..

[36] Luiza Andreazza N., Vevert-Bizet C., Bourg-Heckly G., Sureau F., Jose Salvador M., Bonneau S. (2016). Berberine as a photosensitizing agent for antitumoral photodynamic therapy: Insights into its association to low density lipoproteins. Int. J. Pharm..

[37] Singh I.P., Mahajan S. (2013). Berberine and its derivatives: A patent review (2009–2012). Expert Opin. Biol. Ther..

[38] Vuddanda P.R., Chakraborty S., Singh S. (2010). Berberine: A potential phytochemical with multispectrum therapeutic activities. Expert Opin. Investig. Drugs.

[39] Sun P., Wang T., Chen L., Yu B.W., Jia Q., Chen K.X., Fan H.M., Li Y.M., Wang H.Y. (2016). Trimer procyanidin oligomers contribute to the protective effects of cinnamon extracts on pancreatic beta-cells in vitro. Acta Pharmacol. Sin..

[40] Martins A., Vasas A., Schelz Z., Viveiros M., Molnar J., Hohmann J., Amaral L. (2010). Constituents of Carpobrotus edulis inhibit P-glycoprotein of MDR1-transfected mouse lymphoma cells. Anticancer Res..

[41] He L., Zhao C., Yan M., Zhang L.Y., Xia Y.Z. (2009). Inhibition of P-glycoprotein function by procyanidine on blood-brain barrier. Phytother. Res..

[42] Chen L., Sun P., Wang T., Xu N., Jia Q., Li Y.M., Chen K.X. (2014). Hypoglycemic activity of polyphenol-rich extract from Cinnamomun japonicum Sieb. Chin. Tradit. Patent Med..

[43] Zhao B.X., Sun Y.B., Wang S.Q., Duan L., Huo Q.L., Ren F., Li G.F. (2013). Grape seed procyanidin reversal of p-glycoprotein associated multi-drug resistance via down-regulation of NF-kappaB and MAPK/ERK mediated YB-1 activity in A2780/T cells. PLoS ONE.

[44] Shan Y.Q., Ren G., Wang Y.X., Pang J., Zhao Z.Y., Yao J., You X.F., Si S.Y., Kong W.J., Jiang J.D. (2013). Berberine analogue IMB-Y53 improves glucose-lowering efficacy by averting cellular efflux especially P-glycoprotein efflux. Metabolism.

